# Resilience and Vulnerability: Neurodevelopment of Very Preterm Children at Four Years of Age

**DOI:** 10.3389/fnhum.2020.00219

**Published:** 2020-07-14

**Authors:** Julia M. Young, Marlee M. Vandewouw, Hilary E. A. Whyte, Lara M. Leijser, Margot J. Taylor

**Affiliations:** ^1^Diagnostic Imaging, Hospital for Sick Children, Toronto, ON, Canada; ^2^Neurosciences and Mental Health, SickKids Research Institute, Toronto, ON, Canada; ^3^Department of Psychology, University of Toronto, Toronto, ON, Canada; ^4^Department of Neonatology, Hospital for Sick Children, Toronto, ON, Canada; ^5^Department of Paediatrics, University of Toronto, Toronto, ON, Canada; ^6^Section of Neonatology, Department of Pediatrics, Cumming School of Medicine and Alberta Children's Hospital Research Institute, University of Calgary, Calgary, AB, Canada; ^7^Department of Medical Imaging, University of Toronto, Toronto, ON, Canada

**Keywords:** preterm, magnetic resonance imaging, neurodevelopmental outcome, brain development, ventriculomegaly

## Abstract

Children born very preterm (VPT) are at high-risk for altered brain development and impaired neurodevelopmental outcomes but are not well-studied before school-age. We investigated 64 four-year-olds: 37 VPT children [<32 weeks gestational age [GA]; 22 males; mean GA: 28.8 weeks ± 1.6], 25 full-term (FT) children (12 males), plus two VPT cases with ventriculomegaly and exceptionally resilient outcomes. All children underwent high-resolution structural magnetic resonance imaging and developmental assessments. Measures of brain volume, cortical thickness, and surface area were obtained. Children born VPT demonstrated reduced cerebral and cerebellar white matter volumes yet increased cerebral gray matter, temporal lobe, occipital lobe and ventricle volumes after adjusting for total brain volume. Cortical thickness was greater in the VPT children compared to FT children across all lobes. On developmental assessments, the VPT children scored lower on average than FT children while the two cases had intact cognitive abilities. In addition to larger ventricle volumes, the two cases had white matter and gray matter volumes within the ranges of the FT children. The VPT children displayed distinct differences in structural brain volumes at 4 years of age, consistent with delayed maturation. The cases with persistent ventriculomegaly and good cognitive outcomes displayed typical gray matter and increased white matter volumes, indicating a potential protective developmental phenomenon contributing to their intact cognitive abilities.

## Introduction

Children born very preterm [VPT, <32 weeks gestational age [GA]] are born during a period that is critical for brain development. At the time of VPT birth, important dynamic processes such as neuronal migration, dendritic and axonal arborisation, synaptogenesis, and myelination are ongoing (Kostović and Jovanov-Milosevi, 2006; Lenroot and Giedd, 2006). By term-equivalent age, infants born VPT already display reduced cortical gray matter, white matter, and subcortical volumes and increased cerebral spinal fluid volume, which have been associated with worse neurodevelopmental abilities (Inder et al., 2005; Monson et al., 2016). Differing trajectories of brain development affecting volumes and cortical thickness in VPT children persist into middle and late childhood (Peterson et al., 2000; Kesler et al., 2004; Mürner-Lavanchy et al., 2014; Zhang et al., 2015; Monson et al., 2016).

In addition to altered brain development, VPT infants are at increased risk of brain injury. Up to 30% of very low birth weight preterm infants experience germinal matrix/intraventricular hemorrhage (GMH/IVH) and white matter injury, that can negatively impact neurodevelopmental outcomes (Bolisetty et al., 2014; Mukerji et al., 2015; Holwerda et al., 2016). Post-hemorrhagic ventricular dilatation (PHVD) remains a clinically important complication of particularly large IVH in VPT infants (Brouwer et al., 2014), and has been associated with smaller total brain, deep gray matter and cerebellar volumes as well as poorer neurodevelopmental outcomes (Messerschmidt et al., 2008; Jary et al., 2012; Brouwer et al., 2016). However, some studies in VPT infants with PHVD have found normal neurodevelopmental abilities in the absence of white matter injury (Brouwer et al., 2012; Reubsaet et al., 2017). Therefore, changes in the cerebellum, deep gray matter and cortical white matter may be important determinants for outcome, which has not been fully explored.

Brain development and outcomes are not well-studied in children born VPT before school-age. In addition, protective factors for the rare cases of normal outcome in high-risk VPT cases with persistent ventriculomegaly following early intraventricular hemorrhage (IVH) are not well-understood. The specific aims of this study were to comprehensively describe the structural brain development of VPT children relative to full-term (FT) children at 4 years of age. First, we describe structural measures including volume, surface area, and cortical thickness within cortical, cerebellar and subcortical gray matter as well as cerebral and cerebellar white matter in these two groups. This is the first such detailed comparison at this age. Second, we compare neurodevelopmental outcomes of VPT and FT children. Third, we describe two remarkable, resilient cases from the cohort of VPT children with persistent ventriculomegaly following IVH, and compare their structural brain measures and developmental outcomes to the larger cohort of children born VPT and FT. In addition to investigating differences between structural brain measures and cognitive abilities between children born VPT and FT children at 4 years of age, we explored the structural determinants of positive outcomes despite severe ventriculomegaly.

## Materials and Methods

### Participants

A prospective cohort study was conducted with VPT infants recruited between 2008 and 2010 at the Hospital for Sick Children in Toronto, Canada (REB# 1000047892). Exclusion criteria were any chromosomal or major congenital abnormality. Of the 105 infants born VPT (GA <32 weeks) recruited at birth, five passed away following birth. Fifty-three children returned for follow-up assessments at 4 years of age. Forty-four VPT children acquired both developmental assessments and brain MRI scans. Twenty-seven FT children (GA >37 weeks) were recruited at 4 years of age as controls and underwent developmental assessments and brain MRI scans. Exclusion criteria for FT controls included learning, language, neurological or developmental disabilities and MRI incompatibility. The research ethics board at the Hospital for Sick Children approved the study protocol and all methods were performed in accordance with the relevant guidelines and regulations. Informed written parental consent was obtained and assent was provided by the children at 4 years of age.

### Clinical and Radiological Parameters

Relevant perinatal and neonatal clinical data were obtained for all the VPT children. Pediatric neuroradiologists and a neurologist assessed each of the structural T1- and T2- weighted MR images for the VPT children acquired within 2 weeks of birth. White matter injury and germinal matrix/intraventricular hemorrhage (GMH/IVH) were identified. GMH/IVH was graded according to the classification by Papile and Volpe for cranial ultrasonography findings adapted to MRI (Papile et al., 1978; Volpe, 2008). Two children within the VPT cohort (Case 1 and Case 2) were identified with persistent ventriculomegaly following GMH/IVH during the neonatal period and are described separately from the VPT cohort. Maternal education levels were also obtained.

### Developmental Assessments

Developmental assessments were performed at 4 years of age for the VPT children and FT controls, using standardized tests. Intelligence quotients (IQ) were determined using the Wechsler Preschool and Primary Scales of Intelligence—Third Edition (WPPSI-III) (Wechsler, 2012) applying Canadian norms. Four different cognitive indices were obtained: Verbal IQ (VIQ), Performance IQ (PIQ), Processing Speed (PSQ) and Full-Scale IQ (FSIQ). Overall language ability measuring receptive and expressive language yielding a core language (CL) summary score was determined by the Clinical Evaluation of Language Fundamentals—Preschool, Second Edition (CELF-Pre-2) (Semel et al., 2004). Visual-motor integration (VMI) and supplemental tests of visual perception (VP) and motor coordination (MC) were assessed by the Beery-Buktenica Test of Visual Motor Integration (VMI) (Beery et al., 2010). Raw scores were converted into standardized scores with a population mean of 100 and standard deviation of 15.

### MRI at 4 Years

#### Data Acquisition

MRI scans were acquired on a 3T Siemens Trio scanner and 12 channel head coil. T1-weighted (MPRAGE) anatomical images (repetition/echo time: 2.3/.00296s; field of view: 192 × 240 × 256 mm; resolution: 1 mm^3^; scan time: 5 min) were obtained when the children were awake while watching a movie or naturally asleep. All images were inspected for gross motion artifacts and anatomical abnormalities. Structural images from seven children (5 VPT and 2 FT) were excluded due to excess motion.

#### MRI Processing

**Figure 1:**
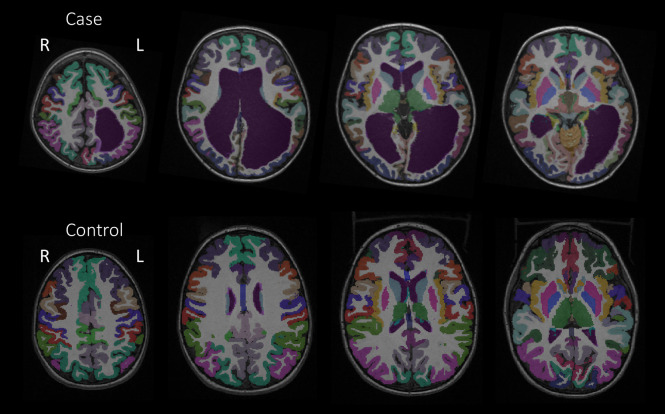
Cortical parcellations from the Desikan-Killiany anatomical atlas of Case 2 and a control to depict anatomical differences.

The Desikan-Killiany anatomical atlas was applied to structural T1-weighted images to segment cortical regions, subcortical regions, white matter, and the cerebellum (34 cortical and 4 subcortical regions bilaterally excluding the brainstem) using Freesurfer 6.0 (Dale et al., 1999; Fischl and Dale, 2000; Fischl et al., 2002; Desikan et al., 2006). After the automatic segmentation, each child's segmentation was assessed for accuracy within Freesurfer. Manual editing of the white matter surface using control points within Freesurfer was applied where necessary as a part of Freesurfer's recommended workflow to ensure accurate segmentations of the gray and white matter. Several iterations of manual editing were performed, and segmentation outputs were thoroughly inspected. All children underwent identical segmentation methods. Frontal lobe measures in three cases and temporal lobe measures in two cases were excluded from the analyses due to uncorrectable segmentations. For the two cases with persistent ventriculomegaly, additional manual editing was required for accurate classifications of the ventricles. Cortical and subcortical parcellations for one of the cases and a control are provided in Figure 1.

Measures of cortical thickness, surface area and volume were calculated for each cortical gray matter region and summed by lobes (frontal, temporal, parietal, and occipital lobes). Cortical measures of volume were calculated for whole brain white matter, cerebellar gray and white matter, as well as subcortical regions. Total brain volume included both gray and white matter volumes combined.

### Statistical Analysis

#### Developmental Measures

Between the VPT and FT children, differences in each of the developmental measures obtained at 4 years of age (VIQ, PIQ, PSQ, FSIQ, CL, VMI, VP, and MC) were assessed using a two-tailed *t*-test. Significance was reported at *p* < 0.05.

#### Structural MRI Measures

Volumes were averaged across left and right hemispheres. Between group differences in absolute volumes and volumes adjusted by total brain volume were analyzed using two-tailed t-tests. Volumes adjusted by total brain volume were calculated by dividing subjects' volumes by their total brain volumes to obtain a ratio. Adjusting by total brain volume helps to account for individual variability in total brain size (Durston et al., 2001; Sowell et al., 2007; Mills et al., 2016). Differences in cortical surface area and cortical thickness by lobe were also adjusted by total brain volume and analyzed using two-tailed *t*-tests. Significance was reported at *p* < 0.05 and corrected for multiple comparisons with an FDR of 5%. Age at scan and total brain volume across groups were analyzed with Pearson correlations.

Structural measures obtained from Cases 1 and 2 were not included in the analyses of the VPT cohort and qualitatively contrasted to the VPT and FT groups. Where the cases displayed absolute volumes that exceeded the ranges of the VPT and FT groups, percentages of volumes compared to the average volumes of the VPT group are described. The statistical analyses and plots were completed in MATLAB (The MathWorks, Inc., Natick, MA, USA) and R Studio.

## Results

### Child Characteristics

Thirty-seven children born VPT (22 males; mean age: 4.22 years ± 0.19) of the original 44 VPT children, independent of the two cases with persistent ventriculomegaly, were included based upon successful structural brain MRI and developmental assessments. Perinatal clinical and radiological characteristics of the VPT children are provided in Table 1. Twenty-five of the recruited 27 FT children were also included (12 males; mean age: 4.53 years ± 0.28).

**Table 1:** Clinical and radiological characteristics of VPT children.

**Characteristic**	**Case 1**	**Case 2**	**VPT group mean (SD)**
Gestational Age (weeks)	31.14	26.57	28.77 (1.61)
Sex	F	M	15 F, 22 M
Birth Weight (g)	1,490	1,050	1129.18 (233.35)
Head circumference (cm)	29	24	25.49 (1.93)
Intrauterine growth restriction	-	-	5 (13.51%)
Cesarean-section delivery	+	+	22 (59.46%)
Multiple births	-	-	7 (17.07%)
Apgar score at 5 min	4	5	7.11 (1.59)
CRIB II	2	11	6.89 (2.34)
Endotracheal tube days	11	17	11.81 (17.28)
Oxygen administration days	10	23	14.22 (23.80)
Positive pressure ventilation	20	19	13.65 (11.31)
Patent ductus arteriosus (treated)	-	-	10 (27.203%)
Sepsis (positive blood culture)	+	+	11 (29.73%)
Premature rupture of membranes	-	+	5 (13.51%)
Necrotizing entercolitis	-	-	5 (13.51%)
Bronchopulmonary dysplasia	-	-	6 (16.22%)
GMH (Grade 1–2)	-	-	7 (17.07%)
GMH (Grade 3–4)	+	+	7 (17.07%)
White Matter lesions	-	-	11 (29.73%)
Maternal education level			*N (%)*
< High school	-	-	1 (2.9)
High school	-	-	6 (17.1)
Post-secondary education	-	+	6 (17.1)
University	-	-	20 (57.1)
Post-graduate education	+	-	3 (8.6)

*CRIB II, Clinical Risk index for Babies; GMH, Germinal Matrix Hemorrhage*.

**Figure 2:**
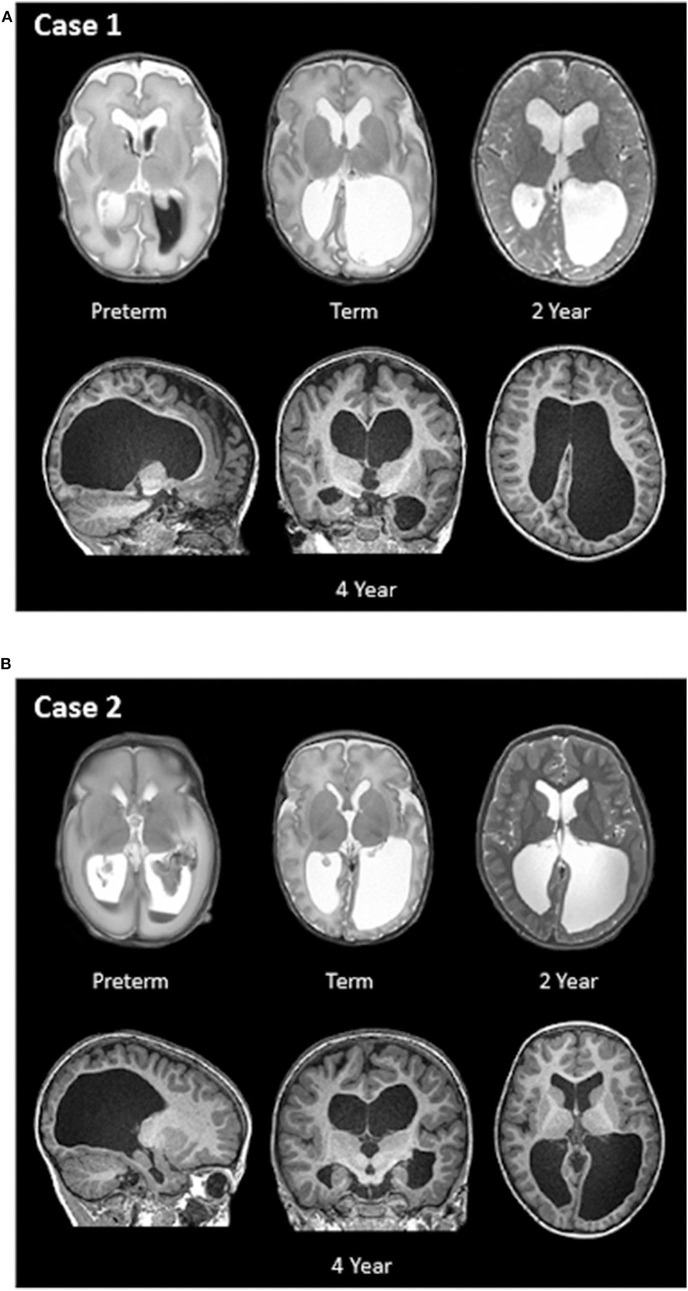
**(A,B)** Longitudinal structural MR images of Case 1 and Case 2. T2- and T1-weighted MRI images were obtained at four different ages: within two weeks of birth (preterm; top left), term-equivalent age (term; top middle), 2 years of age (top right), and 4 years of age (bottom row).

Case 1: This female was born at 31.1 weeks GA with bilateral grade 3 IVH complicated by severe ventricular dilatation seen on MRI 5 days after birth. Longitudinal MR imaging showed persistent ventriculomegaly (Figure 2). Perinatal and neonatal parameters are described in Table 1.

Case 2: This male was born at 26.6 weeks' GA with bilateral grade 3 IVH complicated by severe ventricular dilatation, seen on early ultrasound and confirmed with MRI 14 days after birth. Figure 2 also shows longitudinal MR imaging for this case. Perinatal and neonatal parameters are described in Table 1.

### Developmental Measures

The VPT and FT children performed within the average range compared to the normative sample on most assessments, except for motor coordination in the VPT group (Table 2). When comparing groups, however, the VPT children scored significantly lower than the FT children on all items (Table 2). Case 1 demonstrated scores of PIQ, PSQ, CL, and MC on par with the FT children and higher than the VPT children. Compared to the FT children, VIQ, VMI, and VP were above average. Case 2 similarly demonstrated scores of PIQ, PSQ, CL, and VP in the average range, close to the FT children, with slightly higher scores of VIQ and VMI. Only MC was below average and closer to the scores of the VPT children on that subtest. These two children were followed behaviorally until 8 years of age, and their IQs remained high, being 131 and 116, respectively.

**Table 2:** Developmental assessments at 4 years of age.

**Subjects**	**Value**	**VIQ**	**PIQ**	**PSQ**	**FSIQ**	**CL**	**VMI**	**VP**	**MC**
VPT	Subjects	35	36	30	35	33	36	33	34
	Mean	98.83	94.83	93.33	94.86	94.39	99.14	94.39	85.12
	Std dev	17.05	12.20	17.57	14.00	16.16	10.82	19.97	14.98
FT	Subjects	24	24	20	24	21	24	24	24
	Mean	108.00	105.54	109.45	107.88	110.24	108.92	105.25	96.75
	Std Dev	13.22	13.15	10.69	13.31	12.44	8.90	10.06	8.34
	*p*-value	0.031	0.002	<0.001	<0.001	<0.001	<0.001	0.018	0.001
Case 1	Std score	126	104	109	114	108	115	113	101
Case 2	Std score	115	100	109	107	100	112	106	80

*VIQ, verbal IQ; PIQ, performance IQ; PSQ, processing speed; FSIQ, full scale IQ; CL, core language; VMI, visual motor integration; VP, visual perception; MC, motor coordination; std score, standard score*.

### Structural MRI Measures

#### Differences Between VPT and FT Children

**Figure 3:**
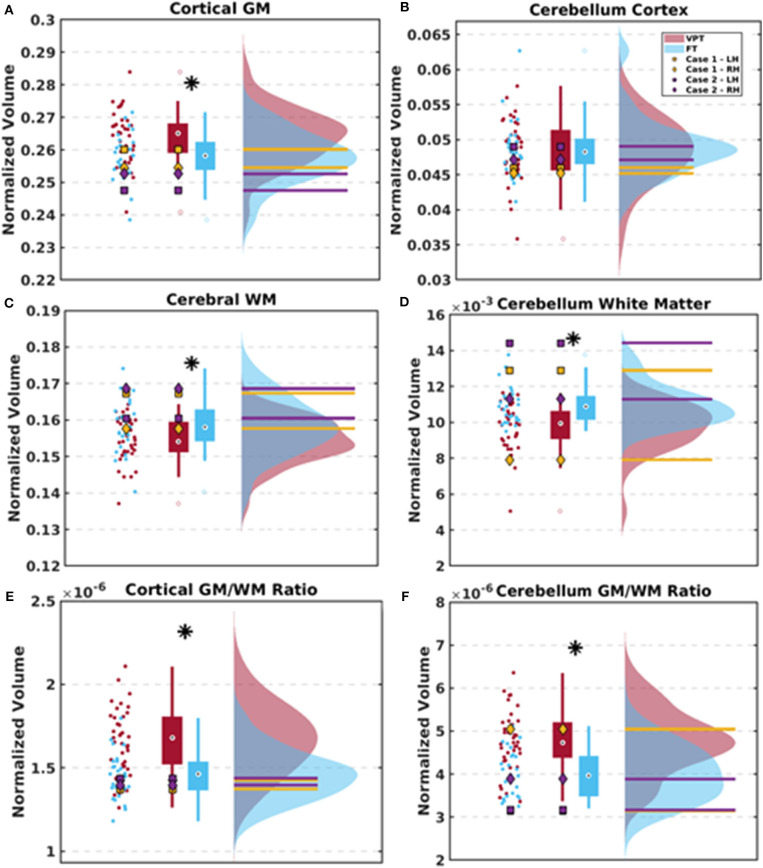
Scatter plots, box plots, and histograms are used to depict the results between the VPT and FT group as well as the two cases. **(A–D)** Group differences between the ratios of cortical gray matter (GM), cerebral white matter (WM), cerebellar GM, and cerebellar WM volumes adjusted for total brain volumes. Data for the VPT and FT control groups are averaged across left and right hemispheres. **(E,F)** Group differences in the ratios of cortical and cerebellar GM to WM after adjusting for total brain volume. Case 1 is depicted in yellow and Case 2 is depicted in purple, with measures separated by left (LH) and right hemispheres (RH) due to some brain asymmetries. * indicates significant group differences following FDR threshold of 5%.

The 4-year-old children born VPT displayed reduced total brain volumes compared to the FT children. After adjusting for total brain volumes, the pallidum and cortical and cerebellar white matter volumes were significantly reduced in the VPT children (Table 3). In contrast, adjusted cortical gray matter, temporal lobe, and occipital lobe volumes were significantly greater in children born VPT (Table 3). The children born VPT also had significantly greater cortical and cerebellar ratios of gray matter to white matter compared to the FT children. The results between the VPT and FT groups are detailed in Table 3 and illustrated in Figures 3A–F. Absolute structural volumes *without* adjusting for total brain volume are reported in Supplemental Table 1. Age at scan was not associated with total brain volume across groups (*p* = 0.19).

**Table 3:** Average volumes adjusted for total brain volume at 4 years of age.

	**Mean ratio (SD)**				
**Structure**	**Case 1**	**Case 2**	**VPT**	**FT**	***p-value***
Total Brain volume (cm^3^)	1132.9	1072.8	1041.3 (113.4)	1128.6 (80)	**0.002**
Ventricle (cm^3^)	192.2	148.4	9.8 (5.9)	6.5 (2.2)	**0.011**
Cortical gray matter	0.2573	0.2501	0.2641 (0.0077)	0.2579 (0.0076)	**0.003**
Cortical white matter	0.1625	0.1645	0.1539 (0.0057)	0.158 (0.0073)	**0.021**
Cerebellar gray matter	0.0455	0.0481	0.0478 (0.0066)	0.0487 (0.0043)	0.488
Cerebellar white	0.0104	0.0129	0.0098 (0.0013)	0.0109 (0.0011)	** <0.001**
Frontal lobe	0.1002	0.0975	0.0999 (0.0051)	0.0972 (0.0041)	0.046
Parietal lobe	0.0719	0.0642	0.0718 (0.0043)	0.0722 (0.0041)	0.59
Temporal lobe	0.0557	0.0558	0.0579 (0.0022)	0.0558 (0.0028)	**0.003**
Occipital lobe	0.0253	0.0283	0.0294 (0.0024)	0.0276 (0.0022)	**0.003**
Thalamus	0.0064	0.0060	0.0059 (0.0009)	0.0059 (0.0003)	0.20
Caudate	0.0033	0.0036	0.0033 (0.0006)	0.0032 (0.0003)	0.52
Putamen	0.0034	0.0042	0.0046 (0.0007)	0.0044 (0.0004)	0.16
Pallidum	0.0012	0.0016	0.0015 (0.0002)	0.0016 (0.0001)	**0.001**

*Bolded p-values significant following FDR threshold of 5%. Note: total brain and ventricle volumes are unadjusted*.

In each lobe, the VPT group demonstrated significantly greater average cortical thickness compared to the FT children (*p* < 0.001; Supplemental Table 2 and Supplemental Figure 1). No lobar differences were found in surface area between groups.

#### Cases With Persistent Ventriculomegaly

Cortical and cerebellar gray matter volumes for Cases 1 and 2 were similar to those of the VPT and FT children while ventricular volumes were 19.5 times larger in Case 1 and 15.1 times larger in Case 2 than in the VPT group (Table 3). Adjusted volumetric measures obtained for Cases 1 and 2 are shown as distinct data points separated by hemisphere within each plot due to differences in their left and right ventricular sizes (Figures 3A–F).

Both Case 1 and 2 had cerebral white matter volumes that were above average compared to the VPT children (115 and 110% the averaged volumes of the VPT group, respectively) and in range with the FT children (Supplemental Table 1). After normalizing for total brain volume, both cases had cerebral and cerebellar white matter volumes in one hemisphere that were greater than the FT children (Figures 3B,D). Cerebellar white matter volumes within the left hemisphere were greater in Case 1 and Case 2 compared to both VPT children (116% and 135% the averaged volumes of the VPT group, respectively) and FT children, on the side of the largest ventricular dilatation (Supplemental Table 1, Figure 3D). Both cases had lobar volumes on par with the VPT and FT children. Case 1 demonstrated more variable subcortical volumes while Case 2 displayed subcortical volumes within the range of the two groups (Supplemental Table 1).

In Case 1, cortical thickness was lower than the average of the VPT and FT children in each lobe except for the frontal lobe. In Case 2, cortical thickness of each lobe was within the range of the FT children (Supplemental Table 2; Supplemental Figure 1). Measures of surface area for both cases were also within the range of both groups in the temporal and occipital lobes. Case 2 had lower surface area in the parietal lobe yet greater surface area in the frontal lobe compared to the FT children (Supplemental Table 2).

## Discussion

In our study of 4-year-old children born VPT and FT, differential patterns of brain development were evident. After adjusting for total brain volume, VPT children had increased total gray matter, temporal and occipital gray matter, and reduced pallidum, cortical white matter, and cerebellar white matter compared to FT controls. The children born VPT also demonstrated poorer cognitive outcomes on average, although the scores were in average range. In contrast, the two exceptional cases with persistent ventriculomegaly displayed an alternate neurodevelopmental profile with greater cerebral and cerebellar white matter volumes, yet gray matter volumes within the range of FT children. This structural profile in the cases combined with very good outcomes may suggest a protective phenomenon against abnormal outcome. This is the first study to provide detailed structural brain measures and neurodevelopmental outcomes in children born VPT at 4 years of age, and to show the potential sparing of gray and white matter volumes for normal outcome despite ventriculomegaly in early childhood.

Previous studies in older VPT children have demonstrated widespread differences in cortical and subcortical brain volumes (Peterson et al., 2000; Zhang et al., 2015; Monson et al., 2016), with VPT children typically displaying smaller volumes. Affected structures included the cerebellum, deep gray matter and regions in the frontal, temporal, and parietal lobes (Peterson et al., 2000; Kesler et al., 2004). Monson et al. (2016) reported that VPT infants displayed less growth in absolute cortical and subcortical gray matter volumes and sustained discrepancies in white matter between term-equivalent age and 7 years of age compared to FT children, suggesting that brain development in children born VPT does not catch up to their FT peers during early and late childhood. Our findings of increased gray to white matter ratios in the VPT children, despite having overall reduced gray and white matter volumes compared to FT controls, extend these results. As gray matter steadily decreases throughout childhood (Mills et al., 2016; Vandewouw et al., 2020), increased gray matter in the VPT group is consistent with delayed maturation. Longitudinal studies in VPT children between late childhood and early adolescence have also documented delayed maturation in gray and white matter volumes and slower gains in white matter compared to FT children (Ment et al., 2009; Nagy et al., 2009), although this does not always continue past adolescence (Karolis et al., 2017).

Cortical thickness was greater in the VPT children compared to the FT across lobes. Cortical thinning is a fundamental process of brain development during childhood and reflects synaptic and neural pruning as well as an increase in myelin penetration to lower cortical layers (Sowell et al., 2004). From 7 to 12 years of age, VPT children were found to have continuing cortical thinning in frontal and parietal regions while cortical thinning was largely complete in these regions by 7 years of age in FT controls, reflecting a potential delay in this process in the VPT children (Mürner-Lavanchy et al., 2014). Thus, our study provides evidence of delayed cortical thinning in the VPT children, indicating divergent maturational trajectories of cortical thickness at 4 years of age.

The underlying neurodevelopment of children with PHVD and ventriculomegaly with normal cognitive outcomes remains largely unclear. Our two cases with persistent ventriculomegaly following early IVH were surprisingly resilient with respect to their structural brain measures at 4 years of age. Their cortical gray and white matter volumes were in the ranges of the FT children while white matter volumes of the left cerebellar hemisphere were increased at the ipsilateral side of the largest ventricle. As such, these findings may provide a potential explanation for their intact cognitive abilities.

Post-hemorrhagic ventricular dilatation (PHVD) and non-hemorrhagic ventriculomegaly due to complications of severe IVH or perinatal illness in VPT infants are known to increase the risk for poorer neurodevelopmental outcomes in childhood, such as motor disabilities and cognitive impairments (Ment et al., 1999; Murphy et al., 2002; Pappas et al., 2017). Infants with PHVD who also have parenchymal injury and/or undergo neurosurgical interventions (e.g., ventriculoperitoneal shunt insertion), combined with anesthesia and risk of related complications, often have poor cognitive, motor, attention, and visual perception outcomes (Brouwer et al., 2014; Holwerda et al., 2016; Leijser et al., 2018). Despite what is typical in infants/children with severe PHVD (Tam et al., 2011; Jary et al., 2012; Morita et al., 2015; Brouwer et al., 2016), our two cases did not lose significant brain volume (Jary et al., 2012).

The absence of white matter lesions, relatively stable neonatal clinical courses and gradual progressions of ventricular dilatation without need for intervention likely contributed to their typical outcomes (Futagi et al., 2005). In addition, frontal and temporal white matter regions may have compensated for the ventricular dilatation primarily within the parietal and occipital regions. The size of the ventricles in the other VPT children were significantly larger than the FT children on average, yet not as extreme as two cases, which could be due to cortical volume loss related to prematurity or white matter injury. While these cases are certainly exceptional, our findings strengthen the hypothesis that the presence of additional white matter lesions and changes in cortical, subcortical and cerebellar volumes may be more important determinants of neurodevelopmental outcomes compared to PHVD or ventriculomegaly alone.

There are some limitations to consider within the present study. Although obtaining MRI scans on children at 4 years of age is challenging due to compliance, the sample size of the studied cohort at 4 years of age is relatively small. As such, the results of the present study would be strengthened with a larger sample size. A longitudinal study of the structural brain measures and cognitive outcomes would provide greater insight into neurodevelopmental differences between the children born very preterm and term-born children. Further, information on other factors such as nutrition, therapy, or other treatments could be helpful in understanding potential influences on neurodevelopmental trajectories. Additional cases of PHVD and persistent ventriculomegaly would also be beneficial for understanding the neurodevelopmental outcomes of these children. Furthermore, cortical white matter was not analyzed by lobe using our methodology and thus we could not comment on white matter volumes within the different lobes.

In conclusion, the present study demonstrates for the first time that at 4 years of age, children born VPT present with brain dysmaturation characterized by increased cortical gray matter and reduced white matter volumes after adjusting for total brain volume, and poorer cognitive outcomes in comparison to FT children. Both structural findings suggest delayed maturation, as cortical gray matter decreases and white matter increases with age. Our two cases with isolated persistent ventriculomegaly following early IVH, displayed typical neurodevelopmental outcomes and age-appropriate brain volumes suggesting that sparing of gray and white matter volumes, and absence of associated lesions may be important determinants of outcome in those with PHVD and persistent ventriculomegaly. Long-term outcome studies in large cohorts of preterm infants with persistent ventriculomegaly are needed to support this hypothesis. Taken together, the overall results demonstrate altered neurodevelopmental outcomes in children born very preterm as well as the possibility for normal neurodevelopmental outcomes in children with isolated, slowly progressing PHVD.

## Data Availability Statement

The datasets generated for this study are available on request to the corresponding author.

## Ethics Statement

The studies involving human participants were reviewed and approved by Research Ethics Board, The Hospital for Sick Children. Written informed consent to participate in this study was provided by the participants' legal guardian/next of kin. Written informed consent was obtained from the minor(s)' legal guardian/next of kin for the publication of any potentially identifiable images or data included in this article.

## Author Contributions

JY was responsible for the study design, analyses, and writing the manuscript. MV was responsible for image and data analysis and reviewing the manuscript. HW was responsible for reviewing the manuscript. LL was responsible for study design, writing, and reviewing the manuscript. MT was responsible for study design and reviewing the manuscript. All authors contributed to the article and approved the submitted version.

## Conflict of Interest

The authors declare that the research was conducted in the absence of any commercial or financial relationships that could be construed as a potential conflict of interest.

## Abbreviations

VPT: very preterm
FT: full term
GA: gestational age
IVH/GMH: intraventricular hemorrhage/germinal matrix hemorrhage
IQ: intelligence
PHVD: post-hemorrhagic ventricular dilatation
VIQ: verbal IQ
PIQ: performance IQ
PSQ: processing speed
FSIQ: full scale IQ
CL: core language
VMI: visual motor integration
VP: visual perception.
