# Supplementary material for: Resilience and Vulnerability: Neurodevelopment of Very Preterm Children at Four Years of Age
Source: Front Hum Neurosci. 2020 Jul 14;14:219. doi: 10.3389/fnhum.2020.00219 (PMC7372104; doi:10.3389/fnhum.2020.00219)

**Supplemental information**

| **Table 1. Average *unadjusted* volumes by group and cases at four years of age** | | | | | |
| --- | --- | --- | --- | --- | --- |
|  | **Mean (SD) Volumes (cm^3^)** | | | |  |
| **Structure** | **Case 1** | **Case 2** | **VPT** | **FT** | ***p-value*** |
| **Cortical grey matter** | 291.5 | 268.3 | 274.9 (31.7) | 290.9 (24.3) | ***0.037*** |
| **Cortical white matter** | 184.1 | 176.5 | 160.6 (18.7) | 178.8 (15.7) | ***<0.001*** |
| **Cerebellar grey matter** | 51.6 | 51.6 | 49.7 (8.1) | 54.9 (4.2) | ***0.004*** |
| **Cerebellar white** | 11.8 | 13.8 | 10.2 (1.8) | 12.4 (1.5) | ***<0.001*** |
| **Frontal lobe** | 113.5 | 104.6 | 104.6 (12.4) | 109.9 (10.8) | *0.096* |
| **Parietal lobe** | 81.4 | 68.9 | 74.5 (8.2) | 81.7 (8.2) | ***0.001*** |
| **Temporal lobe** | 63.1 | 59.9 | 60.6 (6.9) | 62.9 (5.4) | *0.18* |
| **Occipital lobe** | 28.7 | 30.4 | 30.7 (4.6) | 31.1 (2.9) | *0.71* |
| **Thalamus** | 7.2 | 6.4 | 6.1 (0.7) | 6.8 (0.5) | ***<0.001*** |
| **Caudate** | 3.7 | 3.9 | 3.4 (0.6) | 3.6 (0.4) | *0.11* |
| **Putamen** | 3.8 | 4.5 | 4.8 (0.6) | 5.0 (0.5) | *0.076* |
| **Pallidum** | 1.4 | 1.7 | 1.5 (0.2) | 1.8 (0.2) | ***<0.001*** |
| *Bolded p-values significant following FDR threshold of 5%.* | | | | | |

| **Table 2. Cortical Thickness and Surface Area at four years of age** | | | | | |
| --- | --- | --- | --- | --- | --- |
| **Average (SD) Cortical Thickness (1/mm^2^ x 10^-5^)** | | | | | |
| **Structure** | **Case 1** | **Case 2** | **VPT** | **Full-term** | ***p-value*** |
| **Frontal lobe** | 3.62 | 3.72 | 3.87 (0.41) | 3.58 (0.35) | **<.001** |
| **Parietal lobe** | 1.64 | 1.78 | 1.9 (0.21) | 1.77 (0.14) | **<.001** |
| **Temporal lobe** | 2.32 | 2.66 | 2.78 (0.32) | 2.56 (0.28) | **<.001** |
| **Occipital lobe** | 0.75 | 0.87 | 0.96 (0.11) | 0.89 (0.08) | **<.001** |
|  | **Average (SD) Surface Area (1/mm x 10^2^)** | | | |  |
| **Frontal lobe** | 2.6 | 2.77 | 2.74 (0.16) | 2.69 (0.17) | 0.394 |
| **Parietal lobe** | 2.27 | 2.07 | 2.17 (0.15) | 2.19 (0.12) | 0.586 |
| **Temporal lobe** | 1.42 | 1.43 | 1.47 (0.11) | 1.46 (0.12) | 0.976 |
| **Occipital lobe** | 1.03 | 1.04 | 1.01 (0.09) | 0.97 (0.09) | 0.456 |
| *bolded p-values remain significant following an FDR threshold of 5%* | | | | |  |

**Supplemental Fig. 1. Cortical Thickness Results**


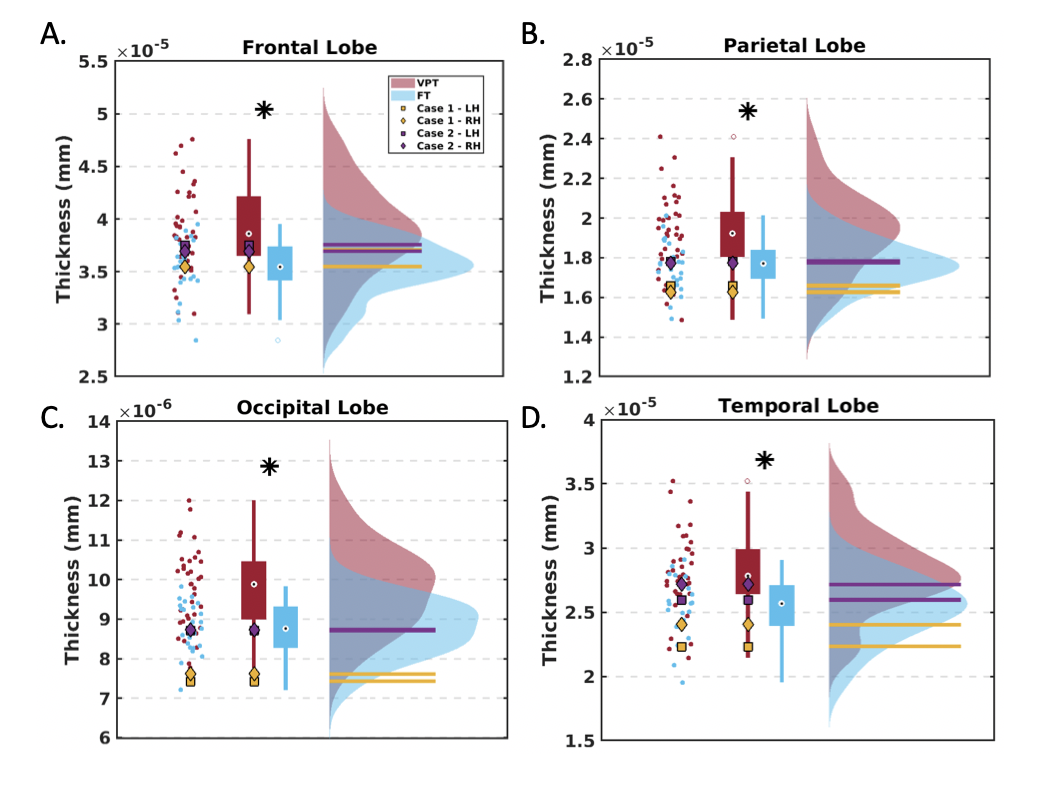

Supplement: Supplementary file 1 [file Table_1.DOCX]
